# Valganciclovir Dosing Strategies for Cytomegalovirus Prophylaxis in Pediatric Solid Organ Transplant Recipients: A Comparative Single-Center Study

**DOI:** 10.3390/v18030297

**Published:** 2026-02-28

**Authors:** Samar Alharbi, Delal Alkortas, Dema Alissa, Aziza Ajlan, Zinah Alabdulkarim, Hala Joharji, Khalid Alhasan, Edward Bentz Devol, Dalia Obeid, Ahmed Al-jedai

**Affiliations:** 1 Organ Transplant Center of Excellence, King Faisal Specialist Hospital and Research Center, Riyadh 11211, Saudi Arabia; aajlan@kfshrc.edu.sa (A.A.); kalhasan28@yahoo.com (K.A.); 2 College of Medicine, Al-Faisal University, Riyadh 11533, Saudi Arabia; dalkortas@gmail.com (D.A.); daalissa@gmail.com (D.A.); zeina_faiek@hotmail.com (Z.A.); hala.joharji@hotmail.com (H.J.); aaljedai@alfaisal.edu (A.A.-j.); 3 College of Pharmacy, Al-Faisal University, Riyadh 11533, Saudi Arabia; 4 College of Medicine, King Saud University, Riyadh 11362, Saudi Arabia; 5 Pediatric Department, King Saud University Medical City, Riyadh 11362, Saudi Arabia; 6 Department of Biostatistics, Epidemiology & Scientific Computing, King Faisal Specialist Hospital and Research Center, Riyadh 12713, Saudi Arabia; edevol@kfshrc.edu.sa; 7 Transplant Research and Innovation Department, Organ Transplant Centre of Excellence, King Faisal Specialist Hospital and Research Centre, Riyadh 11211, Saudi Arabia; dobeid@kfshrc.edu.sa

**Keywords:** cytomegalovirus, valganciclovir, pediatric transplant, prophylaxis, kidney transplant, liver transplant

## Abstract

Optimal valganciclovir dosing for cytomegalovirus (CMV) prophylaxis in pediatric solid organ transplant recipients remains difficult due to variable pharmacokinetics. This study compared the efficacy and safety of three dosing strategies: body surface area (BSA)-based, weight-based, and Pescovitz algorithm-based dosing. This retrospective cohort study included pediatric patients aged ≤14 years who underwent kidney or liver transplantation between 2010 and 2018 and received valganciclovir for CMV prophylaxis. The primary outcome was the comparative effectiveness of the three dosing approaches in preventing CMV infection and disease. The study included 150 patients, with 50 in each group. CMV infection occurred more frequently in the BSA-based group than in the Pescovitz algorithm-based or weight-based groups. Among patients who developed CMV DNAemia, CMV disease occurred in 54%, 28%, and 50% of patients in the BSA-based, Pescovitz algorithm-based, and weight-based groups, respectively. No tissue-invasive CMV disease was observed within 12 months after transplantation. All groups showed acceptable safety profiles, and no patients discontinued therapy due to hematologic adverse events. Weight-based dosing was associated with lower CMV infection and disease rates than BSA-based dosing and Pescovitz algorithm-based dosing, with similar safety. In pediatric solid organ transplant recipients, valganciclovir dosing strategy was not independently associated with CMV infection after adjustment. Age emerged as the sole independent predictor of CMV risk, underscoring the predominance of patient-related factors in CMV prophylaxis.

## 1. Introduction

Cytomegalovirus (CMV) is a common viral infection in pediatric solid organ transplant (SOT) recipients [1]. CMV infection occurs in 30–40% of SOT recipients, and invasive CMV disease develops in 8–30%, depending on the transplanted organ [2]. In immunocompetent individuals, primary CMV infection is often asymptomatic or self-limited, after which the virus establishes lifelong latency with potential for future reactivation [3]. In SOT recipients, CMV infection may occur as primary infection (CMV-positive donor/CMV-negative recipient) or reactivation of latent infection in seropositive recipients, depending on donor–recipient serostatus. These distinct mechanisms influence CMV risk, timing, and clinical presentation following transplantation and form the basis of risk-adapted prophylaxis strategies. Valganciclovir is currently the most widely used agent for CMV prophylaxis in immunocompromised patients, including SOT recipients [4].

The U.S. Food and Drug Administration (FDA) has approved valganciclovir for CMV prevention in children older than 4 months and adults undergoing heart or kidney transplantation. However, dosing in pediatric patients remains challenging due to pharmacokinetic variability. Age, body weight, and creatinine clearance (CrCl) significantly influence dosing. Inappropriate dosing can result in toxicity such as granulocytopenia, anemia, and thrombocytopenia or lead to prophylaxis failure and CMV resistance [5,6,7].

Several dosing strategies have been proposed, including body surface area (BSA)-based, weight-based, and BSA combined with CrCl using the Pescovitz algorithm [8,9,10]. In 2009, the manufacturer recommended the Pescovitz algorithm for infants and young children, with a maximum glomerular filtration rate (GFR) of 150 mL/min/1.73 m^2^. However, in 2010, the FDA warned that this algorithm could result in overdosing in children with low body weight, low BSA, and normal serum creatinine levels [11,12,13]. Demirhan et al. reported that weight-based dosing was associated with lower toxicity compared to BSA-based dosing, without increasing CMV DNAemia rates [14]. Currently, no consensus exists regarding the optimal valganciclovir dosing strategy for CMV prophylaxis in pediatric SOT recipients. Therefore, this retrospective, single-center study aimed to compare the efficacy and safety of three valganciclovir dosing strategies in this population.

## 2. Materials and Methods

### 2.1. Study Design

This retrospective cohort study included pediatric patients aged ≤14 years who underwent kidney or liver transplantation between 2010 and 2018 at King Faisal Specialist Hospital and Research Center, Riyadh, Kingdom of Saudi Arabia, and received valganciclovir for CMV prophylaxis after solid organ transplantation. Patients older than 14 years, those who received non-SOT procedures, those on preemptive rather than universal prophylaxis, and those who did not complete at least 3 months of valganciclovir therapy were excluded.

This study was approved by the Institutional Review Board and Ethics Committee of King Faisal Specialist Hospital and Research Center (reference no. 2191106) and conducted in accordance with institutional policies and the Declaration of Helsinki. Informed consent was waived due to the retrospective design.

### 2.2. Study Endpoints

The primary endpoint was the occurrence of the first episode of CMV infection or CMV disease within 12 months after solid organ transplantation. CMV infection was defined as detectable CMV DNA in blood or plasma by polymerase chain reaction (PCR) with a viral load > 30 IU/mL considered positive, irrespective of clinical symptoms, and CMV disease was defined as CMV infection with attributable clinical manifestations.

For patients who developed CMV infection that subsequently resolved, the initial detection of CMV DNAemia was considered the primary endpoint event, and resolution of viremia or subsequent episodes were not counted as additional primary endpoint events.

The secondary endpoint was safety, assessed by the occurrence of hematologic adverse events, including anemia, leukopenia, and thrombocytopenia, during the prophylaxis period.

### 2.3. Study Parameters

Data extracted from electronic health records included age, sex, weight, body surface area (BSA), body mass index, glomerular filtration rate (GFR), CMV donor and recipient serostatus, immunosuppressive therapy, prophylaxis duration, CMV polymerase chain reaction (PCR) results during the 12-month post-transplant period, and laboratory values.

All SOT recipients received oral valganciclovir once daily starting 3–4 days after transplantation. Prophylaxis duration was risk-based: 6 months for high-risk patients (CMV-negative recipient/CMV-positive donor) and 3 months for intermediate-risk patients (CMV-positive recipient/CMV-positive donor). Valganciclovir prophylaxis was administered according to institutional, risk-based protocols, and most patients received the protocol-defined duration. Occasional deviations occurred in routine clinical practice and reflected individualized clinician judgment in this retrospective cohort. Three dosing strategies were used: weight-based dosing (13–16 mg/kg per day); BSA-based dosing (BSA × 520 mg/m^2^); and Pescovitz algorithm-based dosing (dose = 7 × BSA × creatinine clearance [CrCl], where CrCl (modified Schwartz: mL/min/1.73 m^2^) = [k × height (cm)]/serum creatinine (mg/dL), with k = 0.45 for patients aged <2 years, 0.55 for boys aged 2 to <13 years, 0.55 for girls aged 2–16 years, and 0.7 for boys aged 13–16 years. If CrCl exceeded 150 mL/min/1.73 m^2^, a capped value of 150 was used. Doses were rounded to the nearest 25 mg, with a maximum of 900 mg/day. These dosing strategies were applied during different institutional periods as clinical practice evolved. The selection of dosing approach was influenced by both era and clinician preference, reflecting changes in protocols and patient-specific factors including age, renal function, and clinical status.

CMV definitions followed the Third International Consensus Guidelines on the Management of CMV in SOT (2018) [4], endorsed by the American Society of Transplantation and the CMV Drug Development Forum [15,16]. CMV infection was defined as evidence of viral replication regardless of symptoms. CMV disease was defined as CMV infection with attributable symptoms and was classified as viral syndrome (i.e., fever, malaise, leukopenia, and thrombocytopenia) or tissue-invasive disease (i.e., end-organ involvement). CMV DNAemia referred to the detection of CMV DNA in blood or plasma, independent of clinical symptoms.

CMV viral replication was monitored using quantitative polymerase chain reaction (PCR) testing of blood or plasma. CMV PCR testing was performed periodically as part of routine post-transplant surveillance during the prophylaxis period and additionally when clinically indicated.

### 2.4. Immunosuppression Protocol

All pediatric recipients received triple maintenance immunosuppression consisting of tacrolimus, mycophenolate, and prednisone. Induction therapy included methylprednisolone for all patients. Renal transplant recipients additionally received antithymocyte globulin if they underwent deceased-donor transplantation, had more than four antigen mismatched living donors, had ABO-incompatible transplants, or developed delayed graft function. All remaining kidney and liver transplant recipients received basiliximab induction.

### 2.5. Statistical Analysis

Approximately 50 patients per dosing group were expected based on institutional practice patterns; therefore, sample size was determined pragmatically rather than through formal power calculations. Assuming a CMV infection incidence of approximately 40% based on prior reports, a group size of 50 provided a 95% confidence interval margin of error of approximately ±14%. For the total cohort (*n* = 150), the margin of error was approximately ±8%. This precision-based approach supported the study’s descriptive and exploratory objectives. Statistical analyses included paired or independent *t*-tests, chi-square tests or Fisher’s exact tests, and analysis of variance, as appropriate. Data were reported as mean ± standard deviation. Statistical significance was defined as a two-sided *p*-value < 0.05.

## 3. Results

A total of 150 pediatric SOT recipients were included, comprising 86 males and 64 females, with 50 patients in each dosing group. Baseline characteristics are summarized in Table 1. The mean age differed significantly across groups and was 8.63 ± 4.32 years in the weight-based group, 2.79 ± 2.83 years in the BSA-based group, and 5.24 ± 4.62 years in the Pescovitz algorithm-based group (*p* < 0.0001). Mean body weight and baseline kidney function also differed significantly (*p* < 0.0001 and *p* < 0.004, respectively). Sex distribution did not differ, with approximately 60% males in all groups. Kidney transplantation was performed in 92%, 20%, and 36% of the patients in the weight-based, BSA-based, and Pescovitz algorithm-based dosing groups, respectively, whereas liver transplantation was performed in 8%, 80%, and 64%, respectively. Induction therapy differed significantly among groups, with antithymocyte globulin used in 30%, 2%, and 8%, respectively (*p* < 0.0001). Maintenance immunosuppression did not differ significantly among groups.

The primary endpoint was significantly different among the three dosing groups (Table 2). Overall, 60 patients (39.7%) developed CMV polymerase chain reaction (PCR) positivity. The incidence of CMV viremia differed significantly among dosing strategies (χ^2^ = 9.11, *p* = 0.0105). CMV infection occurred in 24% of patients in the weight-based group, compared to 52% in the BSA-based group (*p* < 0.005) and 44% in the Pescovitz algorithm-based group. The difference between the weight-based and Pescovitz algorithm-based groups was not statistically significant by paired *t*-test or analysis of variance (*p =* 0.08 and *p =* 0.47, respectively). One-way ANOVA revealed a significant difference in CMV mean rank scores across groups (F(2148) = 5.70, *p* = 0.0041). Scores were highest in the BSA-based group (17.5 ± 20.7), followed by the Pescovitz algorithm-based group (13.3 ± 19.0), and were lowest in the weight-based group (5.7 ± 13.0). Tukey–Kramer post hoc analysis confirmed a significant difference between BSA-based and weight-based dosing (*p* = 0.003). These findings were also supported by the Kruskal–Wallis test (χ^2^ = 13.86, *p* = 0.001).

Among patients who developed CMV DNAemia, CMV disease occurred in 50% (6/12) of the weight-based dosing group, 54% (14/26) of the BSA-based dosing group, and 28% (6/22) of the Pescovitz algorithm-based group. No cases of tissue-invasive CMV disease, graft loss, or death occurred during the prophylaxis period.

As shown in Table 2, the mean white blood cell count, hemoglobin, and platelet count during the prophylaxis period were 9.26 ± 4.16 × 10^9^/L, 95.98 ± 16.24 g/L, and 332.28 10 ± 89.11 × 10^9^/L, respectively, in the weight-based dosing group; 12.49 ± 5.54 × 10^9^/L, 93.22 ± 16.93 g/L, and 328.55 ± 108.29 × 10^9^/L in the BSA-based dosing group, respectively; and 12.95 ± 6.31 × 10^9^/L, 92.64 ± 2.39 g/L, and 388.48 ± 15.32 × 10^9^/L in the Pescovitz algorithm-based dosing group, respectively. No significant differences were observed in white blood cell count (χ^2^ = 0.47, *p* = 0.79), platelet count (χ^2^ = 0.91, *p* = 0.63), or hemoglobin level (χ^2^ = 4.31, *p* = 0.12).

The mean prophylaxis duration differed significantly among groups: 3.0 ± 2.27 months in the weight-based group, 6.5 ± 4.01 months in the BSA-based group, and 5.0 ± 4.29 months in the Pescovitz algorithm-based group (*p* < 0.0001).

In univariate analysis, younger age (odds ratio [OR], 0.81; 95% CI, 0.73–0.88; *p* < 0.001), lower mean creatinine (OR, 0.96; 95% CI, 0.93–0.98; *p* < 0.001), and weight-based dosing (OR, 0.29; 95% CI, 0.12–0.67; *p* = 0.005) were associated with lower odds of CMV PCR positivity.

Kaplan–Meier analysis demonstrated differences in time to CMV infection across dosing strategies. Mean time to CMV positivity was 6.8 months in the BSA-based group, 11.1 months in the Pescovitz algorithm-based group, and 9.0 months in the weight-based group. The log-rank test showed a significant difference in CMV-free survival (χ^2^ = 9.14, *p* = 0.0103), consistent with the Wilcoxon test (χ^2^ = 9.22, *p* = 0.0099). CMV-free survival was shortest in the BSA-based group, with a median time to CMV detection of 8.5 months.

In multivariable logistic regression adjusting for age, sex, dosing algorithm, mean creatinine, body mass index, transplant type, and induction therapy, only age remained independently associated with CMV PCR positivity (adjusted OR, 0.85; 95% CI, 0.74–0.97; *p* = 0.017) (Table 3). The association between weight-based dosing and CMV infection was not significant after adjustment (adjusted OR, 0.85; 95% CI, 0.30–2.44; *p* = 0.767).

Overall, dosing strategy was not independently associated with CMV infection in adjusted analyses (*p* > 0.05 for all comparisons).

After conversion to a common mg/kg metric (Supplementary Tables S1 and S2), mean valganciclovir doses differed significantly across groups (Kruskal–Wallis χ^2^ = 86.23, *p* < 1.9 × 10^−19^). Mean doses were 29.6 ± 5.2 mg/kg for BSA-based dosing, 39.6 ± 15.7 mg/kg for the Pescovitz algorithm-based group, and 14.0 ± 3.6 mg/kg for weight-based dosing. Unadjusted logistic regression showed a positive association between dose (mg/kg) and CMV PCR positivity (OR per 1 mg/kg = 1.048; 95% CI 1.022–1.074; *p* = 0.00024). However, after adjustment for age, baseline creatinine, transplant type, and basiliximab induction, this association was not significant (adjusted OR per 1 mg/kg = 1.016; 95% CI 0.984–1.048; *p* = 0.339). In Cox proportional hazards analysis, dose (mg/kg) was not associated with CMV hazard (hazard ratio [HR] per 1 mg/kg = 1.002; 95% CI, 0.981–1.023; *p* = 0.884). Age remained independently protective against CMV infection (adjusted OR per year = 0.83; 95% CI 0.70–0.94; *p* = 0.003). These findings indicate that the apparent dose–response relationship observed in unadjusted models was driven by confounding rather than a true protective effect of higher dosing.

## 4. Discussion

Although several studies have evaluated various valganciclovir dosing algorithms based on weight, BSA, or the Pescovitz algorithm, none have directly evaluated their associations with clinical outcomes. To the best of our knowledge, this is the first study to compare the efficacy and safety of these three prophylactic dosing strategies in a pediatric SOT population. Despite shorter prophylaxis duration and more intensive induction therapy, the weight-based algorithm was associated with a significantly lower rate of CMV infection compared with the other two strategies.

Orit Peled et al. demonstrated that weight-based dosing results in significantly lower valganciclovir doses than the Pescovitz algorithm, with up to a 3.5-fold difference in young children. Similarly, in a retrospective study of infants and young children, Pescovitz algorithm-based dosing produced higher systemic exposure, with 80.8% of patients exceeding target AUC and only 15.4% achieving the therapeutic range of 40–60 µg·h/mL. Vaudry et al. reported comparable findings, showing markedly elevated AUCs in children with low BSA (<0.5 m^2^) and high glomerular filtration rate (GFR > 150 mL/min/1.73 m^2^), with mean AUC values of 64.8 ± 34.7 and 69 ± 36.5 µg·h/mL, respectively [9].

Asberg et al. further demonstrated that the manufacturer-recommended Pescovitz algorithm was associated with systematic overdosing in younger children and underdosing in older children, reflecting age-dependent variability in valganciclovir pharmacokinetics. In their pharmacokinetic analyses, only approximately 20% of patients had valganciclovir exposure within the therapeutic area under the curve (AUC) range in the absence of therapeutic drug monitoring, highlighting the limitations of fixed algorithm-based dosing and supporting the need for individualized dose adjustment.

Although pharmacokinetic studies suggest that Pescovitz algorithm-based dosing provides more accurate systemic exposure, our real-world clinical findings differed. In our cohort, weight-based dosing demonstrated lower interpatient variability and comparable clinical efficacy, despite lower theoretical mg/kg dosing. This disparity may reflect the heterogeneity of pediatric SOT populations, where fluctuating renal function, variable body composition, and evolving clinical conditions limit the reliability of complex formulas. Thus, weight-based dosing may offer more consistent prophylactic exposure in everyday clinical practice. The safety profile of valganciclovir in this study was consistent with prior reports. Vaudry et al. also found oral valganciclovir to be generally well tolerated for up to 100 days in pediatric SOT recipients at risk for CMV disease.

Multiple factors influence valganciclovir dosing in children, including the dosing algorithm, the method used to estimate CrCl, and assay variability in serum creatinine measurement.

In our univariate analysis, younger age, lower mean creatinine, and weight-based dosing were associated with lower odds of CMV PCR positivity. However, after multivariable adjustment, only younger age remained independently associated with reduced CMV risk. This indicates that the apparent benefit of weight-based dosing was largely confounded by age-related differences across groups.

When all regimens were standardized to mg/kg, the Pescovitz- and BSA-based strategies delivered substantially higher doses than the weight-based protocol, reflecting patient selection and clinical practice patterns. The association between higher mg/kg dosing and increased CMV incidence in unadjusted models was eliminated after adjusting for age, renal function, transplant type, and induction therapy. This strongly suggests confounding by indication rather than a true biological dose–response relationship.

Several limitations must be acknowledged. First, the retrospective design introduces potential selection and information bias. The three dosing strategies were implemented during different institutional periods and were associated with differences in CMV risk stratification, age distribution, renal function, induction therapy, and prophylaxis duration. As such, the groups were not directly comparable within a causal framework. Although multivariable adjustment was performed, residual confounding related to correlated patient characteristics and temporal practice changes cannot be excluded. Therefore, the findings should be interpreted as observational and hypothesis-generating rather than as definitive evidence of comparative efficacy. Second, adverse events related to valganciclovir were based on chart documentation and may have underestimated mild or transient toxicities. Third, extension of prophylaxis beyond the protocol-defined duration was based on clinician judgment and patient-specific risk factors, which may have influenced CMV outcomes.

## 5. Conclusions

In pediatric solid organ transplant recipients, valganciclovir dosing strategy was not independently associated with CMV infection after adjustment for clinical confounders. Age emerged as the sole independent predictor of CMV risk, indicating that patient-related factors exert a greater influence on CMV outcomes than dosing algorithm selection. These findings support a risk-adapted approach to CMV prophylaxis and highlight the importance of individualized assessment in pediatric transplant populations.

## Figures and Tables

**Table 1:** Baseline characteristics of pediatric solid organ transplantation recipients receiving cytomegalovirus prophylaxis.

Patient Characteristics	Weight-Based Dosing (*n* = 50)	Body Surface Area-Based Dosing (*n* = 50)	Pescovitz Algorithm-Based Dosing (*n* = 50)	*p*-Value
Age (years) (mean ± SD)	8.63 ± 4.32	2.79 ± 2.83	5.24 ± 4.62	<0.0001
Weight (kg) (mean ± SD)	22.52 ± 10.50	11.31 ± 5.65	17.66 ± 14.50	<0.0001
Height (cm) (mean ± SD)	115.49 ± 25.10	85.01 ± 20.97	96.48 ± 4.24	<0.0001
BSA (m^2^) (mean ± SD)	0.84 ± 0.30	0.53 ± 0.20	0.67 ± 0.37	<0.0001
Male sex, *n* (%)	30 (60%)	28 (56%)	28 (56%)	0.897
Kidney function (modified Schwartz formula) (mean ± SD) mL/min	135.92 ± 57.02	176.51 ± 53.85	163.04 ± 71.44	0.004
CMV serology, *N* (%)				<0.001
Donor positive/recipient negative	0 (0%)	0 (0%)	8 (16%)	
Transplanted organ, *N* (%)				<0.001
Kidney	46 (92%)	10 (20%)	18 (36%)	
Liver	4 (8%)	40 (80%)	32 (64%)	<0.001
Type of organ, *N* (%)				0.608
Deceased donor	9 (18%)	8 (16%)	5 (10%)	
Kidney	9 (100%)	1	0	
Liver	0 (0%)	7	5	
Living donor, *N* (%)	41 (82%)	42 (84%)	45 (90%)	
Kidney	37	10	18	
Liver	4	32	27	
Induction treatment, *N* (%)				
Antithymocyte globulin	15 (30%)	1 (2%)	4 (8%)	<0.001
Basiliximab	35 (70%)	49 (98%)	46 (92%)	<0.001
Methylprednisolone	50 (100%)	50 (100%)	50 (100%)	
Maintenance treatment				
Tacrolimus, *N* (%) ng/mL	50 (100%)	50 (100%)	49 (98%)	
Tacrolimus level at 3 months (mean ± SD)	8.76 ± 1.22	7.84 ± 1.70	8.68 ± 1.52	0.437
Tacrolimus level at 6 months (mean ± SD)	8.37 ± 1.10	7.64 ± 1.53	8.36 ± 1.34	0.321
Tacrolimus level at 12 months (mean ± SD)	8.07 ± 1.5	7.41 ± 1.42	8.36 ± 1.30	0.505
Cyclosporine, *N* (%) ng/mL	0	0	1 (2%)	
Cyclosporine level at 3 months (mean ± SD)			233.6	
Cyclosporine level at 6 months (mean ± SD)			227.25	
Cyclosporine level at 12 months (mean ± SD)			179.86	
Mycophenolate, *N* (%)	39 (78%)	44 (88%)	49 (98%)	0.289
Mycophenolate/m^2^/dose (mean ± SD)	361.31 ± 194.47	407 ± 160.98	208.37 ± 133.77	
Azathioprine, *N* (%)	8 (16%)	6 (12%)	1 (2%)	0.430
Azathioprine/m^2^/dose (mean ± SD)	54.34 ± 35.08	42.45 ± 11.63	40	
Sirolimus	0	0	0	
Everolimus	0	0	0	
Prednisone or prednisolone, *N* (%)	50 (100%)	50 (100%)	50 (100%)	0.149
Prednisone or prednisolone/m^2^/dose (mean ± SD)	7.28 ± 5.92	8.43 ± 7.99	5.91 ± 3.67	
Prophylaxis duration, months (median ± SD)	3 ± 2.27	6.5 ± 4.01	5 ± 4.29	<0.0001

**Table 2:** Efficacy and safety of valganciclovir cytomegalovirus prophylaxis in pediatric solid organ transplantation recipients.

	Weight-Based Dosing (*n* = 50)	Body Surface Area-Based Dosing (*n* = 50)	Pescovitz Algorithm-Based Dosing (*n* = 50)	*p*-Value
Positive CMV PCR (viremia), *n* (%)	12 (24%)	26 (52%)	22 (44%)	0.013
Mean CMV viral load,	1048.27	1299	3331	<0.001
CMV disease, *n* (%)	6 (12%)	14 (28%)	6 (12%)	0.007
Tissue-invasive disease, *n* (%)	0	0	0	
Graft loss, *n* (%)	0	0	0	
Death, *n* (%)	0	0	0	
Platelet count 10^9^/L (mean ± SD)	332.28 ± 89.11	328.55 ± 108.29	388.48 ± 15.32	0.0095
Hemoglobin g/L (mean ± SD)	95.98 ± 16.24	93.22 ± 16.93	92.64 ± 2.39	0.6855
White blood cells 10^9^/L (mean ± SD)	9.26 ± 4.16	12.49 ± 5.54	12.95 ± 6.31	0.0025

**Table 3:** Univariate and multivariate logistic regression predicting CMV-positive results by patients’ clinical characteristics.

Characteristic	Univariate Models			Multivariate Model		
	OR1	95% CI1	*p*-Value	OR1	95% CI1	*p*-Value
Age (years)	0.81	0.73, 0.88	<0.001	0.85	0.74, 0.97	0.017
Sex						
Female	—	—		—	—	
Male	0.68	0.35, 1.32	0.25	0.72	0.34, 1.51	0.384
Dosing algorithm used						
Body surface area (520 mg/m^2^ × BSA)	—	—		—	—	
Pescovitz algorithm-based (7 × BSA × CrCl)	0.73	0.33, 1.59	0.42	1.32	0.55, 3.24	0.539
Weight-based (13–16) mg/kg)	0.29	0.12, 0.67	0.005	0.85	0.30, 2.44	0.767
Creatinine	0.96	0.93, 0.98	<0.001	0.98	0.95, 1.00	0.189
Body mass index	0.94	0.84, 1.04	0.25	1.01	0.88, 1.15	0.901
Type of transplant						
Deceased	—	—		—	—	
Living	0.56	0.22, 1.41	0.22	0.60	0.18, 1.84	0.374
Basiliximab used	1.80	0.68, 5.32	0.25	0.56	0.14, 2.30	0.412

OR, odds ratio; CI, confidence interval.

## Data Availability

All data and materials are available upon request.

## Supplementary Materials

The following supporting information can be downloaded at: https://www.mdpi.com/article/10.3390/v18030297/s1, Dose conversions and dose-response analyses. Table S1: Valganciclovir dose converted to mg/kg according to dosing algorithm (BSA-based, Pescovitz algorithm-based, and weight-based); Table S2: Logistic regression analysis evaluating predictors of CMV PCR positivity (unadjusted and adjusted models); Figure S1: Boxplot of valganciclovir dose (mg/kg) by dosing algorithm.

## Abbreviations

The following abbreviations are used in this manuscript:

BSA	Body surface area
FDA	Food and Drug Administration
GFR	Glomerular filtration rate
PCR	Polymerase chain reaction
