# Supplementary material for: Valganciclovir Dosing Strategies for Cytomegalovirus Prophylaxis in Pediatric Solid Organ Transplant Recipients: A Comparative Single-Center Study
Source: Viruses. 2026 Feb 28;18(3):297. doi: 10.3390/v18030297 (PMC13030751; doi:10.3390/v18030297)
Supplement: Supplementary file 1 [file viruses-18-00297-s001.zip › viruses-4035995-supplementary.pdf]

Supplementary Material: Dose conversions and dose-response analyses

Supplementary Methods:
All valganciclovir doses were converted to mg per kg for each patient. For the BSA-based protocol, total dose was calculated as 520 mg/m² × BSA. For Pescovitz algorithm (7 × BSA × CrCl) was used; CrCl was approximated by the Modified Schwartz formula provided in the dataset. For weight-based regimens the administered dose was divided by patient weight. Dose quartiles were derived from the distribution of mg/kg actual and used for Kaplan–Meier analyses. Time intervals for time-to-event were mapped to median months of the interval (1–3→2 months; 3–6→4.5 months; 6–9→7.5 months; 9–12→10.5 months).

Table S1. Valganciclovir dose converted to mg/kg according to dosing algorithm (BSA-based, Pescovitz algorithm-based, and weight-based).

Dosing Algorithm	*n*	Mean (mg/kg)	SD	Median	IQR	Range (25th-75th)
Body surface area	50	29.62	5.19	30.0	5.95	26.55 - 32.51
Pescovitz algorithm	50	39.61	15.7	45.38	25.56	26.64 - 52.21
Weight-based	50	14.05	3.57	13.85	4.4	11.57 - 15.98
Overall	150	27.75955070708865	14.334015770883147	26.41547049441786	21.58	15.345571218177993 - 36.92307692307692

Kruskal–Wallis $\chi^2 = 86.23$, $p < 1.9\times10^{-19}$.

Table S2. Logistic regression analysis evaluating predictors of CMV PCR positivity (unadjusted and adjusted models).

Model	Variable	Coef	OR (95% CI)	p-value
Unadjusted	mg/kg (per 1 mg/kg)	0.0466	1.048 (1.022 - 1.074)	0.0002
Adjusted	mg/kg (per 1 mg/kg)	0.0154	1.016 (0.984 - 1.048)	0.3389
Adjusted	Age (per year)	-0.1815	0.834 (0.739 - 0.942)	0.0033
Adjusted	Serum creatinine (μmol/L)	-0.0059	0.994 (0.977 - 1.011)	0.4917

Adjusted	Basiliximab induction (yes vs no)	-0.4594	0.632 (0.164 - 2.437)	0.5048

Cox proportional hazards for time to CMV (n = 54): HR per 1 mg/kg = 1.002 (95% CI 0.981–1.023), p = 0.884.

Figure S1. Boxplot of valganciclovir dose (mg/kg) by dosing algorithm.

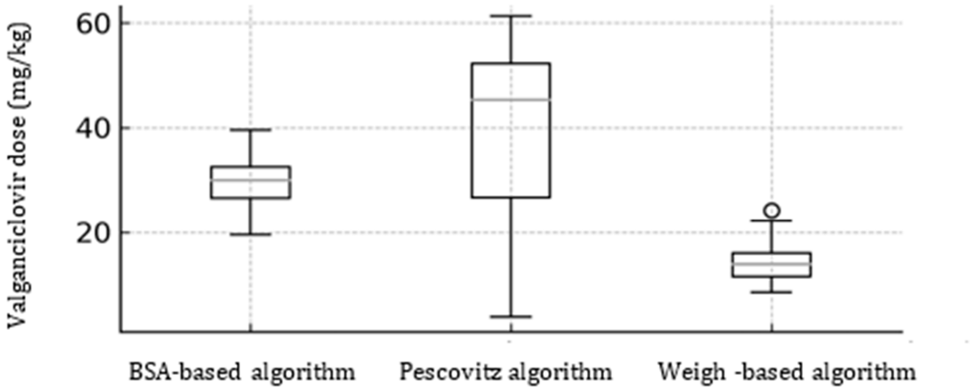

Figure S1 legend: Boxplot of valganciclovir dose (mg/kg) by dosing algorithm. Median and IQR shown; whiskers indicate 5th–95th percentiles.
